# Effect of environmental enrichment and group size on the water use and waste in grower-finisher pigs

**DOI:** 10.1038/s41598-021-95880-0

**Published:** 2021-08-12

**Authors:** Shilpi Misra, Eddie A. M. Bokkers, John Upton, Amy J. Quinn, Keelin O’Driscoll

**Affiliations:** 1 Pig Development Department, Animal and Grassland Research and Innovation Centre, Teagasc, Moorepark, Co. Cork, P61 C996 Ireland; 2 Animal Production Systems Group, Wageningen University and Research, P.O. Box 338, 6700 AH Wageningen, The Netherlands; 3 Livestock Systems Department, Animal and Grassland Research Innovation Centre, Teagasc, Moorepark, Co. Cork, P61 C996 Ireland

**Keywords:** Environmental impact, Animal behaviour

## Abstract

The grower-finisher stage accounts for 64% of the total on-farm herd water use. Part of this is consumed by the pigs, but a part is also wasted. Drinking water usage and wastage is affected by different factors. We investigated how different group sizes and different levels of enrichment affect water usage (ingested plus wasted), water wastage, behavior and performance in grower-finisher pigs. Pigs (n = 672), 11 weeks of age (77 ± 2 days) were used for the experiment. The effect of group size: SMALL (12 pigs), MEDIUM (24 pigs), and LARGE (48 pigs) was assessed across two levels of enrichment (LOW—wooden post, hanging rubber toy, HIGH—Same as LOW + fresh grass). There was no effect of group size on water use or wastage. Pigs with HIGH enrichment (10.4 ± 0.4 L/pig/day) used less water than LOW enrichment (11.0 ± 0.4 L/pig/day; *p* < 0.001). The water wastage/drinker/hour was lower in pens with HIGH enrichment than LOW (*p* = 0.003). The drinking bout number (*p* = 0.037) and total occupancy/hour (*p* = 0.048) was also higher for pens with LOW than HIGH enrichment. Aggressive and harmful behaviour were performed less in LARGE groups and pens with HIGH enrichment. Thus, HIGH enrichment allowance reduced water usage and wastage so may have benefits for the environment, as well as animal welfare.

## Introduction

Global demand for meat products is likely to increase as both world population and incomes grow^1^. This might lead to a surge in the demand for pork, one of the most globally eaten meats^2^. Pig production already contributes 19% to the global water footprint of farm animal production^3^. This means about 6000 m^3^ of water per ton of pigmeat^3^ of which 70% is used off-farm for the production of feed, 24% is used on-farm for drinking, cleaning and feed mixing, and 6% for slaughtering^4^. As global freshwater supplies are limited, it is of major importance that water use in the pig production chain is optimized.

Provision of sufficient water for drinking is considered fundamental in animal agriculture to ensure good welfare. In pig production, drinking water accounts for 80–87% of the total on-farm water use^5,6^ and the grower-finisher stage accounts for 64% of the total herd water use^5^. During this stage, drinking water use ranges from 1.9 to 6.8 L/pig/day^7^. Part of this water is indeed consumed by the pigs, but a part is also wasted. Besides impacting on fresh water resources, water wastage increases the volume of the slurry which dilutes the nutrient content. This increases the operating costs (i.e. cost for manure processing and disposal), and is therefore another reason for minimising water wastage.

Drinking water usage and wastage, and the ratio between the two, is affected by a complex interaction of both pig (e.g. body weight, social competition, feed intake)^8,9^, environmental (e.g. temperature, humidity)^10^ and management factors (e.g. drinker type, pen design)^7,8,11^. An important factor which affects drinking behavior is resource allowance. Researchers found that over a period of 24 h groups of 10 growing pigs had more visits to the single nipple drinker at night than groups of 3^12^. This was hypothesized to result from a higher proportion of interrupted visits during the day, as there was likely more competition for access to the drinker in the larger groups during periods when pigs are normally active. Group size appears to affect both water use and drinking behavior; this was also found in a study where pigs in groups of 20 engaged more with the drinker (more visits to the drinker, and spent more time drinking) but used less drinking water than pigs in groups of 60^8,13^. When stocking density is kept constant, pigs in larger groups have more shared space per pig, which could provide a more complex and engaging environment for them. The impact of this on drinking behavior has not been fully explored, but it could be that more shared space leads to less engagement with the drinker resulting in less wastage, as pigs have a greater area for exploration.

These results also indicate that pigs do not seem to use drinkers purely for drinking^8,13^. The barren environment in which pigs typically live could also promote the performance of redirected (foraging) behavior. This can manifest itself in the form of playing with drinkers^9,14^ which leads to water wastage. Providing appropriate environmental enrichment can reduce the occurrence of these kinds of negative behaviors^15^ and thereby has the potential to reduce water wastage. The use of a range of enrichment materials suitable for slatted systems has been compared, and it was found that loose material provided in a rack was preferred by pigs over point source items such as wooden chew bars, rubber toys etc.^16^. With regard to the type of material to provide in a rack, either fresh grass, or grass silage seems favored by pigs over straw; silage keeps pigs occupied for longer^17^, and more grams of fresh grass are used per day than straw^15^.

To reduce water wastage it is essential to understand drinking behavior, and water wastage in relation to this. Although we know that across all the production stages, grower-finisher pigs consume most of the total water used on a farm and several studies have focused on water use of grower/finisher pigs^7,8,11–13,18,19^ no study has focused on the impact of group size and the effect of enrichment on both drinking behavior and water wasted from drinkers. Therefore, the objective of this study was to investigate how different group sizes and different levels of enrichment affect water usage (ingested plus wasted), water wastage, behavior and performance in grower-finisher pigs. We hypothesized that a larger group size and provision of a favored enrichment material will optimize water use by reducing waste.

## Methods

### Experimental facilities

The study was conducted in the Teagasc Pig Research Facility in Ireland, a farrow to finish experimental pig unit with a 200 sow herd. The experiment was carried out from July 2019 to April 2020. Ethical approval was obtained from the Teagasc Animal Ethics Committee (TAEC233-2019); all procedures were carried out in accordance with the Irish legislation (SI no. 543/2012) and the EU Directive 2010/63/EU for animal experiments.

### Animals, housing, diet and husbandry

A total of 672 pigs [Danish Duroc × (Large White × Landrace)] were included in the experiment. Between weaning (4 weeks of age) and the start of the experiment (11 weeks of age; 77 ± 2 days) pigs were managed in weaner pens (2.4 m × 2.6 m) of 12 pigs. The experiment was carried out over three replicates in a single room. The room contained 4 pens for each experimental group size: SMALL (4.2 m × 2.5 m; 12 pigs), MEDIUM (5.0 m × 4.2 m; 24 pigs), and LARGE (8.2 m × 5.0 m; 48 pigs), providing a space allowance of 0.86 m^2^ per pig in all treatments (Room layout in Supplementary material S1). The room also contained two hospital pens which were of the same size as the pens of the SMALL treatment.

The pigs were fed *ad-libitum* with a standard pelleted diet (43.5% wheat, 30% maize, 17.1% soya-HIpro, 7.1% soya hulls) through single space wet/dry feeders with a built-in nipple. Pigs could mix the water and feed in the trough as required. The feed intake per feeder was recorded through a computerized feeding system (BigFarmNet Manager, Big Dutchman Ltd. v3.1.5.51039, Calveslage, Vechta, Germany). Each pen also had a separate nipple in a bowl drinker mounted 35 cm above ground level and 30 cm from the wet/dry feeder. SMALL pens had 1 feeder and 1 separate drinker, MEDIUM pens had 2 feeders and 2 separate drinkers and LARGE pens had 4 feeders and 4 separate drinkers to ensure the same number of pigs per drinker and feeder in each pen. All pens were fully slatted concrete flooring, the room had mechanical ventilation with a roof fan at the center of the room and artificial light was provided 8 h/day. The average room temperature was maintained at 20 °C.

### Treatments and experimental design

The experiment was carried out by using a 3 × 2 factorial design. The effect of group size (SMALL, MEDIUM, LARGE) was assessed across two levels of enrichment (HIGH, LOW). Pigs on the LOW enrichment received one wooden post and one hanging rubber toy/12 pigs. Pigs on the HIGH enrichment received the same, with the addition of one rack/12 pigs containing fresh grass, which was attached to the wall of the pen. All pens were equipped with enrichment materials prior to entry of the pigs.

The day before the commencement of the experiment pigs were weighed individually, then the individual weights within each weaner pen summed. For the SMALL treatment, 6 pigs from two separate weaner pens were mixed. For the MEDIUM treatment two weaner pens of pigs were mixed, and for the LARGE treatment 4 weaner pens were mixed. The final overall average pen weight was 33.8 ± 3.6 kg. There were an equal number of male and female pigs in each of the final groups. Males and female pigs are kept in the same pen in Ireland and this is a common practice in Irish commercial farms. In Ireland, entire males are produced so the slaughter weights are lower to avoid the boar taint problems.

A total of 24 pens were used in the whole experiment, 12 pens in the first replicate and 6 pens each in second and third replicate. The first replicate had 2 pens for each group size and enrichment combination (e.g. 2 SMALL pens with LOW enrichment, 2 MEDIUM pens with LOW enrichment, 2 SMALL pens with HIGH enrichment, 2 MEDIUM pens with HIGH enrichment etc.). In the second and third replicate, we included 1 pen for each group size and enrichment combination (e.g. 1 SMALL pen with LOW enrichment, 1 MEDIUM pen with LOW enrichment, 1 SMALL pen with HIGH enrichment, 1 MEDIUM pen with HIGH enrichment etc.) due to a scarcity of pigs available for enrolment in the study. For replicates 2 and 3 separate halves of the room were used, so that all pens were used twice over the entire experiment.

### Environmental measurements

Daily measurements (logging interval every 15 min) of temperature and humidity were recorded using data loggers (Tinytag, Sussex, UK) set up in the room at 2 m above floor height. All the data loggers were installed in the passages of the room with each passage having 2 data loggers. For the first replicate 6 data loggers were installed in the room, for the second and third replicate 4 data loggers were installed. Before starting the experiment light intensity in each pen (in front of the drinker) was measured using a luxmeter (ISO-TECH, ILM 1337 Light Meter, UK).

### Enrichment measurements

The wood and hanging rubber toy were weighed at the beginning and at the end of the experiment to estimate the rate of wear by the pigs. Loose fresh cut grass (10–20 cm length) was added to the rack at 90 g/pig and was replenished whenever the quantity dropped to below half of the total allowance per rack as described previously^16^.

### Recording and sampling of water usage and wastage

At the beginning of the experiment, water flow rate from each nipple drinker was measured. Water was collected for 30 s in a plastic bag and the volume measured using a 1000 ml graduated cylinder. The average water flow rate was 1.47 ± 0.14 L/30 s (mean ± s.d.). Water meters were installed in all pens, and each one covered one wet/dry feeder and the bowl drinker next to it. Water use was monitored via an automated online water monitoring system (Carlo Gavazzi Automation, Italy). Data was recorded every 15 min.

To record water wasted from each drinker, a wooden box (0.9 × 0.43 × 0.22 m) was designed (Supplementary material l S2, S3). The box surrounded the drinker on all sides, with an opening (0.25 m wide × 0.35 m high) through which the pigs could access the drinker. The opening was positioned 0.35 m above floor level, and there was unrestricted access to the drinker. Water overflow from each drinker was collected using a container (3.6 L capacity) placed inside the box and underneath the drinker, which fitted comfortably to the sides of the box; thus any waste water could not escape between the side of the container and the box. The amount of wasted water was measured one day per week for six weeks, starting 5 days after the pigs were moved into the pens (i.e. 82 ± 2 days old). Once a week (on Monday), between 0930 and 1600 h the amount of water in the container was measured using a 1000 ml graduated cylinder at least once per hour, and more often if necessary.

### Direct behaviour observations

Pig behaviour was directly observed once per week (on Wednesday) starting a week after the pigs were moved into the pens (84 ± 2 days old). For the first two replicates observations were carried out for the entire 9 weeks of the fattening period, but for the third replicate observations were only carried out for the first 5 weeks (interrupted due to COVID-19). Five minutes of continuous all occurrence observation were conducted 4 times a day for each pen at approximately 1000 h, 1100 h, 1400 h and 1500 h. The order of observations for the pens was randomised across each observation time, so that the average time of observation was similar for all treatments. We were primarily interested in performance of damaging behaviours and enrichment use so these behaviours were recorded using the ethogram in Table 1.

**Table 1:** Ethogram of pig behaviour.

	Behaviour	Description
Aggression	Fight	Mutual pushing parallel or perpendicular, ramming or pushing of the opponent with the head, with or without biting in rapid succession and/or head thrusting. Lifting the opponent by pushing the snout under its body
	Bite	Pig bites another pig with a vigorous movement of the head: mouth open, contact made with body
	Head knock	As for threat but makes contact with head against recipient pigs body
Harmful	Tail bite	Pig forcefully bites down on another pigs tail—often reflected in reaction (vocalisation, fast movement away) by the recipient pig
	Ear bite	Pig takes another pigs ear in its mouth and closes jaws around it—often associated with vocalisation or swift head movement to extract its ear by recipient pig
	Belly nosing	Vigorous nosing of another pigs belly when lying
	Bite other	Biting aimed at another part of the body e.g. leg. Not to be confused with aggression
Enrichment	Interacts with wood	Pig bites or touches the wood or its holder with its mouth
	Interacts with hanging toy or chain	Pig bites or touches the hanging toy or chain with its mouth
	Interacts with grass rack	Pig bites or touches the grass or its holder with its mouth

### Video recordings

Video cameras (2.0MP fixed wide angle bullet cameras with 40 m infrared night vision (HIKVision, China) were installed directly over the drinkers on day 25 of the experiment, when pigs were approximately 102 ± 2 days old. All the cameras were directed towards the drinkers and each drinker had a separate camera which continuously recorded for a 24 h period. Data were downloaded onto a 1 TB Hard drive (PC PRO Computers Ltd., Ireland). Preliminary analysis of the water use data (from the water meters) indicated that the drinkers were most in use between 0800 and 2000 h. An hour of video footage was extracted for each drinker (1000–1100 h) for observation and the time taken start to end of visit was also noted. Occupancy of the drinkers and bouts were determined by recording every time a pigs head entered the box around the drinker (snout disappeared within the opening of the box), and the time that the head was removed. From these data the number of bouts, the duration of each bout, and the duration of occupancy per hour were calculated. The identity or sex of the pigs was not recorded.

### Animal performance

Pigs were weighed individually using a digital scale (R323, Rinstrum, Langenfeld, Germany) the day before the trial started, and at the end of the trial period (147 days), and from these weights the average daily gain (ADG) was calculated. From the computer records of feed delivered to each feeder, the total amount of feed delivered to each pen on each day of the trial was calculated so that the average daily feed intake (ADFI) of pigs in the pen could be calculated. From this the average feed conversion ratio (FCR) per pen was calculated. Records were kept of pigs removed from the trial due to injury, illness or death.

### Statistical analysis

All data were analyzed using SAS version 9.4 (SAS Institute Inc., Cary, NC, USA). Prior to analysis the data were examined to visualize the distribution (PROC UNIVARIATE). The water use (ingested plus wasted), water wastage, animal behavior, drinking behavior and performance data were analyzed using linear mixed models (PROC MIXED). All the models included group size (i.e. SMALL, MEDIUM and LARGE), enrichment level (i.e. HIGH and LOW), day and replicate and relevant interactions (group size*enrichment) as fixed effects and pen was included as a random effect.

### Water use and waste

Several different measures of water use were analyzed. First, the total amount of water delivered through each meter was summed to provide a total amount of water delivered per day to each pen. These data were then used in analysis to compare water use over the entire experimental period across treatments. Day was included as a repeated effect.

Following this, a second analysis was carried out which considered only measurements taken between 0930 and 1600 h on the days which waste water was measured. In addition to water wasted, the total water usage and the water ingested (total less waste) were compared during this period. For some of the drinkers, the daily waste water measurements were unavailable due to water overflow from the container. Therefore, the unit for analysis was the drinker, rather than the pen. The model included group size (i.e. SMALL, MEDIUM and LARGE), enrichment level (i.e. HIGH and LOW), drinker, week, replicate and relevant interactions (group size*enrichment) as fixed effects and drinker with pen was included as a random effect. Week within replicate was included as a repeated effect.

The performance of aggressive, harmful, and enrichment directed behaviour summed up for each pen during each recording session, then divided by number of pigs to calculate the rate of performance/pig/session. The average of all sessions within each recording day was then calculated for analysis. As before, the model included group size, enrichment level, week, replicate and relevant interactions (group size*enrichment) as fixed effects and pen was included as a random effect. Week within replicate was included as a repeated effect.

Three parameters were measured for drinking behavior; the number of bouts, the duration of each bout, and the duration of drinker occupancy per hour. The model for number of bouts and the duration of occupancy per hour included drinker within pen as a repeated effect. The model for the duration of each bout included each pig visit to the drinker as a repeated effect.

The model for animal performance (ADG, ADFI and FCR) included group size (i.e. SMALL, MEDIUM and LARGE), enrichment level (i.e. HIGH and LOW), replicate and relevant interactions (group size*enrichment) as fixed effects and pen was chosen as a random effect.

Interactive effects are reported where they occur. Residuals were checked graphically to ensure that the assumptions of the analyses were met. For all analyses, statistical significance was established at α ≤ 0.05. In all cases, the Tukey–Kramer least squares means adjustment for multiple comparisons was used to separate the treatment means.

## Results

### Ambient temperature and humidity

The temperature ranged from 18.3 to 24.5 °C for replicate 1, 15.2–26.0 °C for replicate 2 and 15.8–24.4 °C for replicate 3. The relative humidity ranged from 52.6 to 97.3% for replicate 1, 51.8–94.2% for replicate 2, 46.3–91.5% for replicate 3. The light intensity ranged from 43 to 201 ± 39.7 (lux).

### Overall water usage (ingested plus wasted)

**Figure 1:**
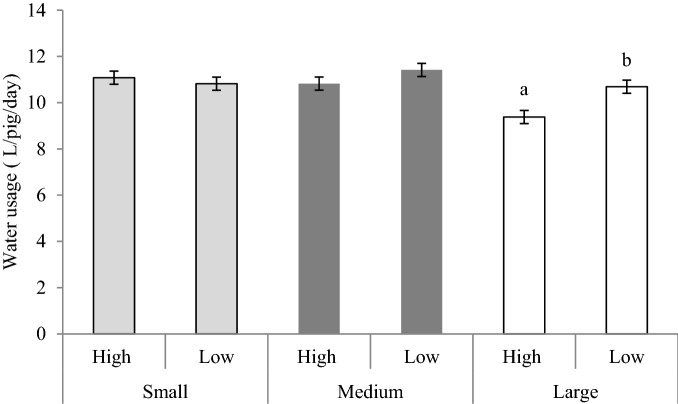
Effect of group size and enrichment on the water usage (ingested + wasted) L/pig/day (LSmeans ± SE). Group size: Large (48 pigs/pen), Medium (24 pigs/pen), Small (12 pigs/pen), Enrichment: Low (Wood + Hanging toy), High (Wood + Hanging toy + Grass). ^abc^Different letters denote significant differences picked up by Tukey–Kramer test (*p* < 0.05).

Pens with HIGH enrichment used less water (10.4 ± 0.4 L/pig/day) than pens with LOW enrichment (11.0 ± 0.4 L/pig/day). There was also an interaction between group size and enrichment (F_1, 138_ = 18.78, *p* < 0.001; Fig. 1). In LARGE groups, those with HIGH enrichment used less water than those with LOW enrichment (*p* < 0.001). A tendency (*p* = 0.083) towards lower water use was also found in MEDIUM pens with HIGH enrichment compared to MEDIUM pens with LOW enrichment.

### Water usage, ingested and wasted from 0930 to 1600 h

The volume of water usage (ingested plus wasted) per drinker per hour was not affected by group size (F_2, 26.7_ = 1.17, *p* = 0.326) or enrichment (F_1, 246_ = 2.32, *p* = 0.129; Table 2). The water ingested (water usage minus wasted) per drinker per hour was not affected by group size (F_2, 26.7_ = 0.76, *p* = 0.477) or enrichment (F_1, 246_ = 0.99, *p* = 0.320). More water was wasted per drinker per hour in pens with LOW enrichment compared to pens with HIGH enrichment (F_1, 61.6_ = 9.82, *p* = 0.003) and there was a tendency towards more water wastage in MEDIUM compared to LARGE pens (F_2, 23.2_ = 3.23, *p* = 0.077). The percentage of water wasted was not affected by group size (F_2, 27.3_ = 0.68, *p* = 0.513) but more water was wasted in pens with LOW enrichment compared to pens with HIGH enrichment (F_1, 74.7_ = 6.46, *p* = 0.013). No interaction was found between group size and enrichment.

**Table 2:** LS means (SEM) of the effect of group size and enrichment on the water usage, water ingested and water wasted from 0930 to 1600 h.

	Treatments						
	Group size				Enrichment		
	Small	Medium	Large	*p* value	High	Low	*p* value
Water usage* (L/drinker/hour)	7.95 (0.90)	8.29 (0.62)	7.18 (0.43)	0.326	7.54 (0.43)	8.07 (0.42)	0.129
Water ingested (L/drinker/hour)	7.03 (0.82)	7.33 (0.57)	6.50 (0.40)	0.477	6.79 (0.40)	7.11 (0.39)	0.320
Water wasted (L/drinker/hour)	0.92 (0.13)	0.94 (0.09)	0.70 (0.06)	0.058	0.76 (0.06)^a^	0.95 (0.06)^b^	0.003
Water wasted (% of the volume wasted)	9.31 (1.19)	8.28 (1.13)	7.53 (1.09)	0.513	7.44 (1.09)^a^	9.36 (1.09)^b^	0.013

Group size: Large (48 pigs/pen), Medium (24 pigs/pen), Small (12 pigs/pen). Enrichment: Low (Wood + Hanging toy), High (Wood + Hanging toy + Grass).

^abc^Different letters denote significant differences picked up by Tukey–Kramer test (*p* < 0.05).

*Water usage includes ingested plus wasted.

### Diurnal pattern of drinking behavior

**Figure 2:**
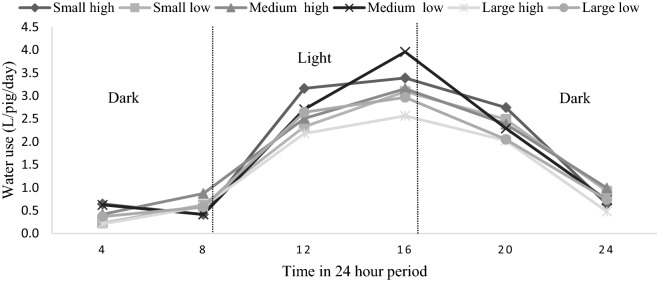
Diurnal pattern of drinking for the six combinations of group size and enrichment (water use/pig/day (L)) occurring during 4-h blocks.

The 24 h period of water use data collected from the water meters of each pen for the entire experimental period was segmented into six blocks of 4 h (logging interval 15 min). The water use (L/pig/day) during each block and for each treatment is shown in (Fig. 2). The diurnal pattern was similar for all the treatments and the water use at the drinkers increased from approximately 8am up to 4 pm and then started declining.

### Frequency and duration of drinker visits

**Figure 3:**
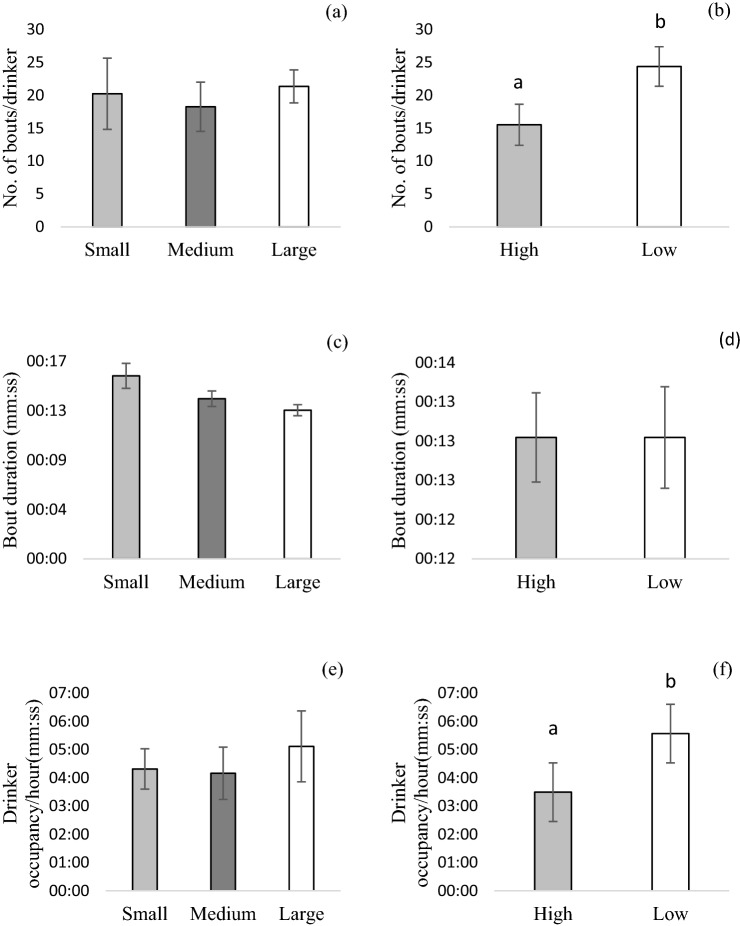
Effect of group size and enrichment on drinking behavior (LSmeans ± SE) (**a**, **b**) number of bouts per drinker (**c**, **d**) duration of each bout (**e**, **f**) occupancy of each drinker per hour. Group size: Large (48 pigs/pen), Medium (24 pigs/pen), Small (12 pigs/pen), Enrichment: Low (Wood + Hanging toy), High (Wood + Hanging toy + Grass) ^abc^Different letters denote significant differences picked up by Tukey–Kramer test (*p* < 0.05).

Pigs in different group sizes had a similar number of drinking bouts but the number of drinking bouts was higher for pens with LOW enrichment (24.4 bouts/drinker/hour) compared to HIGH enrichment (15.5 bouts/drinker/hour, F_1, 18.6_ = 5.08, *p* = 0.037) (Fig. 3a,b). However, the bout duration tended to get shorter as group size increased (F_2, 11.9_ = 3.07, *p* = 0.084) while enrichment had no effect on bout duration (Fig. 3c,d). The total duration of occupancy per hour was not affected by the group size but there was an effect of enrichment (F_1, 18.6_ = 5.08, *p* = 0.048). Pigs in pens with LOW enrichment spent more time occupying the drinker, compared to the pigs with HIGH enrichment (*p* < 0.05) (Fig. 3e,f).

### Animal behavior

The effects of group size and enrichment on pig behavior are presented in Table 3. There was an effect of group size (F_2, 8.34_ = 16.69, *p* = 0.001) and enrichment (F_1, 30.9_ = 9.28, *p* = 0.005) on the amount of aggressive behavior performed by the pigs. Pigs in MEDIUM (*p* = 0.007) and LARGE (*p* = 0.001) groups performed less aggressive behavior than pigs in SMALL groups. Pigs with LOW enrichment performed more aggressive behavior than those with HIGH enrichment (*p* = 0.0047).

**Table 3:** LS means (SEM) of the effect of group size and enrichment on the average number of occurrences of aggressive, harmful, interaction with enrichment behaviours performed per 5-min observation of pens of pigs.

	Treatments													
	Group size				Enrichment			Group size*enrichment						
	Small	Medium	Large	*p* value	High	Low	*p* value	Small		Medium		Large		*p* value
								High	Low	High	Low	High	Low	
Aggression (frequency/pig/recording session)	0.22 (0.02)^a^	0.11 (0.02)^b^	0.07 (0.02)^b^	0.0012	0.11 (0.01)^a^	0.16 (0.01)^b^	0.0047	NI	NI	NI	NI	NI	NI	NI
Harmful (frequency/pig/recording session)	0.36 (0.01)^a^	0.20 (0.01)^b^	0.11 (0.01)^c^	< 0.001	0.20 (0.01)^a^	0.25 (0.01)^b^	< 0.001	0.3 (0.02)^a^	0.41 (0.02)^b^	0.2 (0.02)	0.2 (0.02)	0.08 (0.02)	0.14 (0.02)	0.0066
Interaction with enrichment (frequency/pig/recording session)	0.31 (0.02)^a^	0.31 (0.02) ^a^	0.22 (0.02)^b^	0.033	0.31 (0.01)^a^	0.25 (0.01)^b^	< 0.001	0.34 (0.02)^a^	0.27 (0.02)^b^	0.35 (0.02)^a^	0.26 (0.02)^b^	0.23 (0.02)	0.21 (0.02)	0.071

Group size: Large (48 pigs/pen), Medium (24 pigs/pen), Small (12 pigs/pen), Enrichment: Low (Wood + Hanging toy), High (Wood + Hanging toy + Grass).

*NI* Not included in the final model *p* > 0.10.

^abc^Different letters denote significant differences picked up by Tukey–Kramer test (*p* < 0.05).

Pens with HIGH enrichment performed less harmful behaviour than pens with LOW enrichment (Table 3), but because there was an interaction between group size and enrichment (F_2, 46.3_ = 5.62, *p* = 0.007) these data must be interpreted with caution. In the SMALL groups, pigs provided with LOW enrichment performed more harmful behavior than pigs with HIGH enrichment (*p* < 0.001). Although there was numerically more harmful behaviour performed in pigs with LOW enrichment in the LARGE groups, this difference was not significant (*p* = 0.15). The amount performed in MEDIUM groups was numerically the same regardless of whether enrichment level was LOW or HIGH.

Pigs in LARGE groups interacted less with the enrichment compared to pigs in MEDIUM and SMALL groups (*p* = 0.05), and there was more interaction with enrichment in general in the HIGH enrichment treatment (F_1, 59.8_ = 21.8, *p* < 0.001). There was only a tendency for an interaction between group size and enrichment (F_2, 59.8_ = 2.76, *p* = 0.071) with SMALL and MEDIUM group sizes provided with HIGH enrichment having more interactions with enrichment than LARGE.

### Animal performance

Table 4 summarizes the effect of group size and enrichment on animal performance. Group size had an effect (F_2, 18_ = 4.46, *p* = 0.027) on ADG with LARGE groups having lower ADG than SMALL (*p* = 0.021). ADFI was also influenced by the group size (F_2, 6.52_ = 4.77, *p* = 0.053), again with LARGE groups having lower ADFI than SMALL (*p* = 0.047). The level of enrichment or the interaction between group size and enrichment had no effect on either ADG or ADFI. There was no effect of group size or interaction between group size and enrichment on FCR. However, there was a tendency for pigs on the LOW enrichment treatment to have a lower FCR (*p* = 0.065).

**Table 4:** Effect of group size and enrichment on animal performance (LSmeans ± SEM).

	Treatments						
	Group size				Enrichment		
	Small	Medium	Large	*p* value	High	Low	*p* value
ADG (g/d)	982.4 (17.5)^a^	943.6 (17.5)^ab^	909.9 (17.5)^b^	0.027	944.0 (14.4)	946.5 (14.4)	0.901
ADFI (g/d)	2253.2 (42.8)^a^	2135.7 (42.8)^ab^	2071.9 (42.8)^b^	0.053	2180.7 (34.7)	2126.5 (34.7)	0.284
FCR	2.29 (0.03)	2.26 (0.03)	2.28 (0.03)	0.773	2.31 (0.02)	2.25 (0.02)	0.061

Group size: Large (48 pigs/pen), Medium (24 pigs/pen), Small (12 pigs/pen) Enrichment: Low (Wood + Hanging toy), High (Wood + Hanging toy + Grass).

^abc^Different letters denote significant differences picked up by Tukey–Kramer test (*p* < 0.05).

## Discussion

We hypothesized that a larger group size and provision of a favored enrichment material will optimize water usage by reducing waste. The results indicate that group size did not affect the water usage per pig. However, our hypothesis was confirmed regarding the provision of enrichment; pens with HIGH enrichment used less water per pig than pens with LOW enrichment and this was likely due to less water being wasted in this treatment. HIGH enrichment provision in a larger group size was associated with a reduced rate of performance of aggressive and damaging behaviors, indicating better welfare. Although in the current study pen size and group size were confounded, it is likely primarily the effect of having a larger pen, and more shared space, which drove the effects which were observed.

Water usage includes both water ingested by the animal and water wastage. None of the treatments affected the volume of water usage from 0930 to 1600 h which was 7.81 L/drinker/hour on average. Approximately 89.1% of the total water usage was ingested (6.95 L/drinker/hour) and 10.9% (0.85 L/drinker/hour) was wasted. The amount of water ingested per pig was higher and wastage per pig was lower than other studies (70% ingested and 30% wasted^11^, and > 30% wasted/pig/day)^12^. Drinker design, height and flow rates affect both parameters substantially^7^ and may explain the difference. The design of the drinkers in the current study meant that water which was not consumed by the pig collected in the bowl positioned under the nipple which might have led to less spillage, and this water being drunk by pigs, meaning there was less manipulation of the nipple. Moreover, in our study pigs could also drink from nipples present in the wet/dry feeders which might have influenced the total water usage and wastage, because water that is spilled from these drinker is leaking into the feeder and will be consumed together with the feed and therefore is not wasted. It should also be noted that we had some missing values for water wastage which were excluded from the calculations and this might further affect the results.

The diurnal pattern of water use over a 24 h period in our study is in alignment with previous work. We found the greatest levels of water use during the day with a peak at 1600 h, and a decline in the evening and night. This is similar to the pattern observed by other researchers^8,20^ and it was also reported that the maximum time spent at the drinkers was between 1800 and 1900h^8^. This pattern seems to also follow the typical diurnal feeding pattern of pigs^20^.

Our findings that time spent drinking, and the number of visits to drinkers were not affected by group size, are similar to those of other researchers^12^.The results, however, contradict somewhat with those of other researchers who found that pigs in groups of 20 visited the drinkers more frequently, for longer duration and spent longer time drinking compared to those in groups of 60 pigs; however these studies had varying pigs-to-drinker ratio (10:1 vs 20:1), which likely influenced the results^8,13^. We do acknowledge that our detailed observations of drinking behaviour were only carried out on one occasion during the experiment, and it is possible that the pattern of drinker use may have been different either earlier or later on during the finisher stage. Nevertheless, the results from our analysis of drinker occupancy are in line with the water use and wastage data collected over several weeks; pigs with LOW enrichment had more drinking bouts, greater occupancy of the drinker, and wasted more water both in volume and as a percentage of water usage.

Thus our study provides novel insight into the relationship between enrichment provision, water usage and drinking behavior. We provided grass as additional enrichment in the HIGH treatment as this is highly favorable to pigs, preferred even over other attractive enrichment materials such as shredded paper, and soft wood (e.g. spruce)^15,16^. It seems that part of the pigs’ motivation to forage might have been fulfilled after playing with or consuming grass. They may thus have been less likely to interact with drinkers to satisfy the need to forage, and to interact with them without consuming water. An alternative hypothesis is that grass consumption could have somewhat satiated the pigs thirst; the moisture content of fresh grass is approx. 80%^21^. Thus the pigs may have been less motivated to visit the drinkers for the purpose of drinking and therefore also had less time to spill water. If this were the case, it is possible that our results are somewhat specific to provision of grass as an enrichment material; further research with other enrichment strategies should be performed to determine if the results are consistent across materials. However, it is important to note that the proportion of water which was wasted was higher in the LOW enrichment pens than in the HIGH enrichment. If the amount of water wasted has a linear relationship with the amount used for drinking, we would expect the proportion wasted to be similar across treatments. The fact that the proportion wasted was higher in the LOW treatment indicates that these pig spilled more water when interacting with the drinkers than the pigs in the HIGH treatment.

Nevertheless, the difference in water use across enrichment treatments also varied with group size. There was no effect of enrichment in MEDIUM and LARGE groups. In LARGE pens pigs have more shared space, and therefore more area is available for exploration, and this might have reduced the amount of redirected exploratory behavior toward the drinkers. Moreover, provision of grass as well as shared space had an additive effect, in reducing overall water use.

We found that pigs in SMALL groups had a higher incidence of aggressive and harmful behavior compared to those in MEDIUM and LARGE groups. Our results are not comparable to most studies of group size, because all pigs had equal access to resources in the current study, which was not the case in most other studies. For example, a study investigating the effect of group size along with variation in feeder spaces (1:10 pigs and 1:20 pigs) on the welfare of finishing pigs found that as the group size increased skin lesions, an indication of the amount of aggression, also increased^22^. Pigs from larger group sizes (80 pigs) also tend to be less aggressive when mixed with unacquainted pigs compared with pigs from smaller groups (20 pigs)^23^. The probability of monopolizing resources declines as group size increases, and as the number of competitors increases with group size, fewer individuals get involved in costly fights^24^. Thus, in larger groups pigs appear to become less aggressive and may shift to a low-aggressive social strategy^25^. Increased area of solid flooring and increased space allowance has also been associated with fewer tail damaging behaviors and better overall welfare in finishing pigs^26^.

Although the effect of group size and pen size are confounded in the current study, we thought that it was prudent not to alter what is likely the greater confounding effect of group size and stocking density, which would have occurred if we had not altered the pen size. Moreover, keeping the pen size the same would have meant that the smaller groups would have been managed at a stocking density which would have been approximately 4 times lower than that in typical commercial systems; thus although the results would be theoretically interesting, they would not reflect commercial reality. We must also take into account that the stocking densities across all treatments were slightly lower than that of the EU legal requirement. However the space allowance used is part of the standard operating procedure in the research center, to minimize the risk of damaging behaviors, while remaining somewhat similar to commercial reality. We consider that the level of access to pen resources (enrichment items and feeders) likely has a greater impact on performance of these behaviors than the relatively minor diversion from the legal stocking density limit. A greater number of pigs per feeder may have increased the risk of tail biting or aggression to the point where the results would not reflect typical conditions on a well-managed commercial unit.

Providing sufficient substrate to stimulate foraging and exploratory behavior has been recommended to reduce damaging behavior in pigs^27^. From the current study we conclude that providing pigs with HIGH enrichment reduces aggression and harmful behavior. Our results are in line with^28^, who concluded that pigs in an enriched environment (peat and straw) spent more time on exploratory behavior of substrates than pigs in a barren environment. In this study pigs in a barren environment spent more time in harmful behavior such as nosing and biting, and aggressive behavior such as head thrusting. Moreover, a recent study suggests that pigs with low enrichment replacement rates (i.e. enrichment materials were only replaced every second day after they had been depleted) also perform more aggressive and damaging behavior compared to pig where enrichment replacement rates were more frequent^16^. Thus, in our study the continuous presence of fresh grass as a substrate to explore and eat, likely helped in reducing aggression and harmful behavior. However, it should be noted that our behavioral observations does seem a very short time period, and that there could be a risk of relatively low occurrence of some of the behaviors, and indeed this is why we clustered the behaviors observed into the categories ‘aggression’, harmful’, and ‘enrichment’.

In our study, pigs in LARGE groups had lower feed intake and lower weight gain compared with pigs in SMALL groups, although the feed conversion ratio was similar. This could be because pigs in large groups, usually the dominant pigs, control the access to the feeders^22^. A decline in diurnal variation in feeder visit and feed consumed per hour as group size increased was also reported by other studies^29^. However if feed resources and space allowance are adequate productivity is not affected by group size^30^. Moreover, in the current study, there was a variation in feeder use, likely due to the pen design and the position of the feeders within the pen. Pigs in LARGE pens preferred certain feeders over others. Since all the feeders were not used equally it might have affected the *ad-libitum* feeding behavior of pigs and thus the overall feed intake and weight gain.

## Conclusion

In this study we found no effect of group size on water usage or wastage but pigs with HIGH enrichment had lower water usage and wastage compared to pigs with LOW enrichment. Aggression and harmful behavior was lower only in LARGE groups and in pens with HIGH enrichment not in all group sizes. The pigs in LARGE groups had lower feed intake and daily gains compared to SMALL groups while pig/feeder ratio was equal in all pens. Thus we conclude that providing enrichment to pigs in the form of fresh grass next to wood and hanging toys reduces water usage and wastage and has beneficial effects for welfare.

## Supplementary Information

Supplementary Information.

## Data Availability

The datasets generated during and/or analyzed during the current study are available from the corresponding author on reasonable request.
