# Supplementary material for: Effect of environmental enrichment and group size on the water use and waste in grower-finisher pigs
Source: Sci Rep. 2021 Aug 12;11:16380. doi: 10.1038/s41598-021-95880-0 (PMC8361099; doi:10.1038/s41598-021-95880-0)
Supplement: Supplementary file 1 — Supplementary Information. [file 41598_2021_95880_MOESM1_ESM.pdf]

Effect of environmental enrichment and group size on the water use and waste in grower-finisher pigs

Shilpi Misra[1,2*], Eddie A.M. Bokkers[2], John Upton[3], Amy J. Quinn[1], Keelin O'Driscoll[1]

[1]Pig Development Department, Animal and Grassland Research and Innovation Centre, Teagasc, Moorepark, Co. Cork, P61 C996, Ireland

[2]Animal Production Systems group, Wageningen University & Research, Wageningen, PO Box 338

6700AH, the Netherlands

[3] Livestock Systems Department, Animal and Grassland Research Innovation Centre, Teagasc Moorepark, Co. Cork, P61 C996, Ireland

*shilpi.misra@teagasc.ie

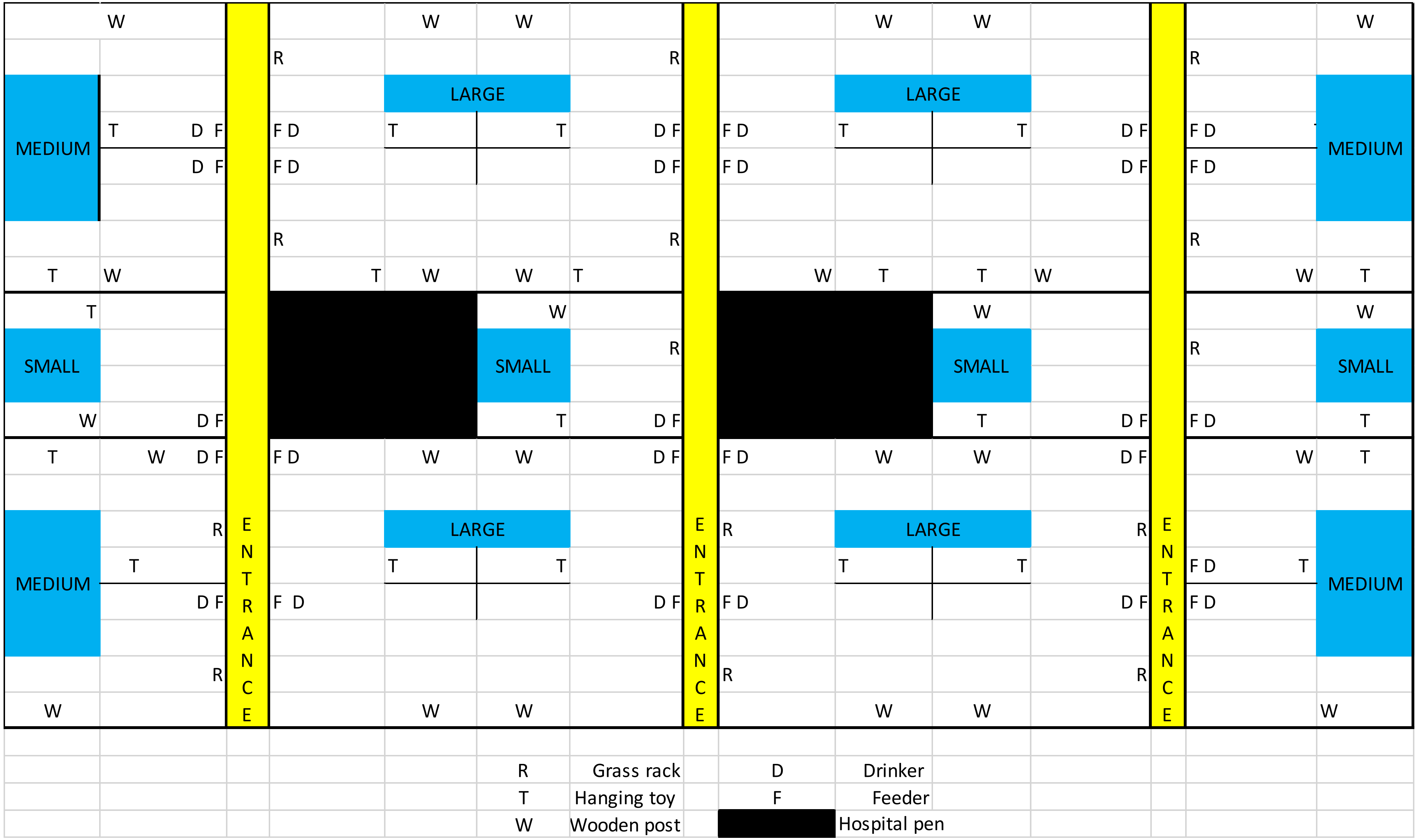

Supplementary Figure S1. Layout of the room housing pigs in group sizes LARGE (48 pigs), MEDIUM (24 pigs) and SMALL (12 pigs) and showing the position of the drinker, feeder and enrichment materials. The black pens were empty and used as hospital pens.

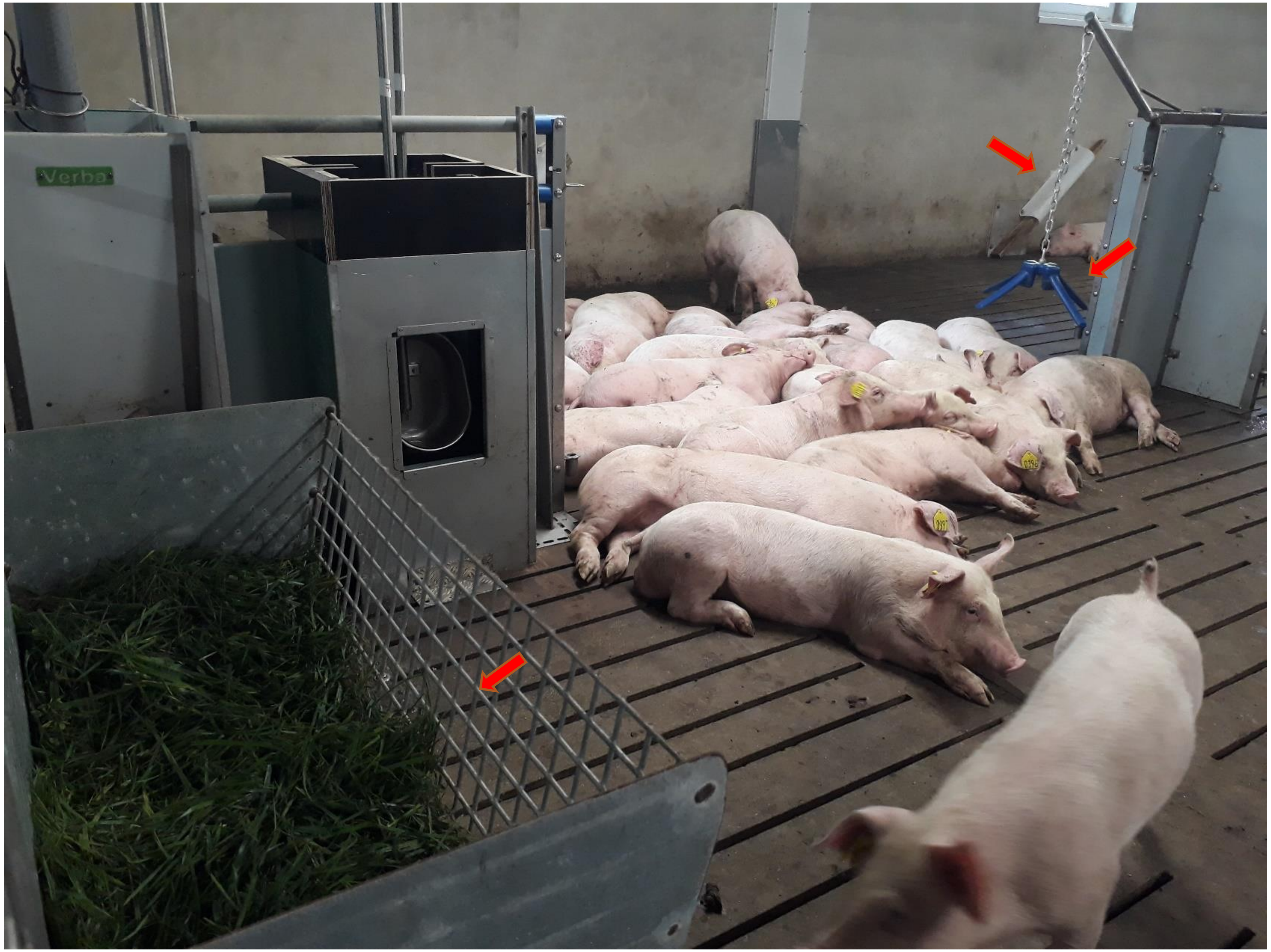

Supplementary figure S2: LARGE pen with wooden box around the drinker, grass rack, hanging toy and wooden post.

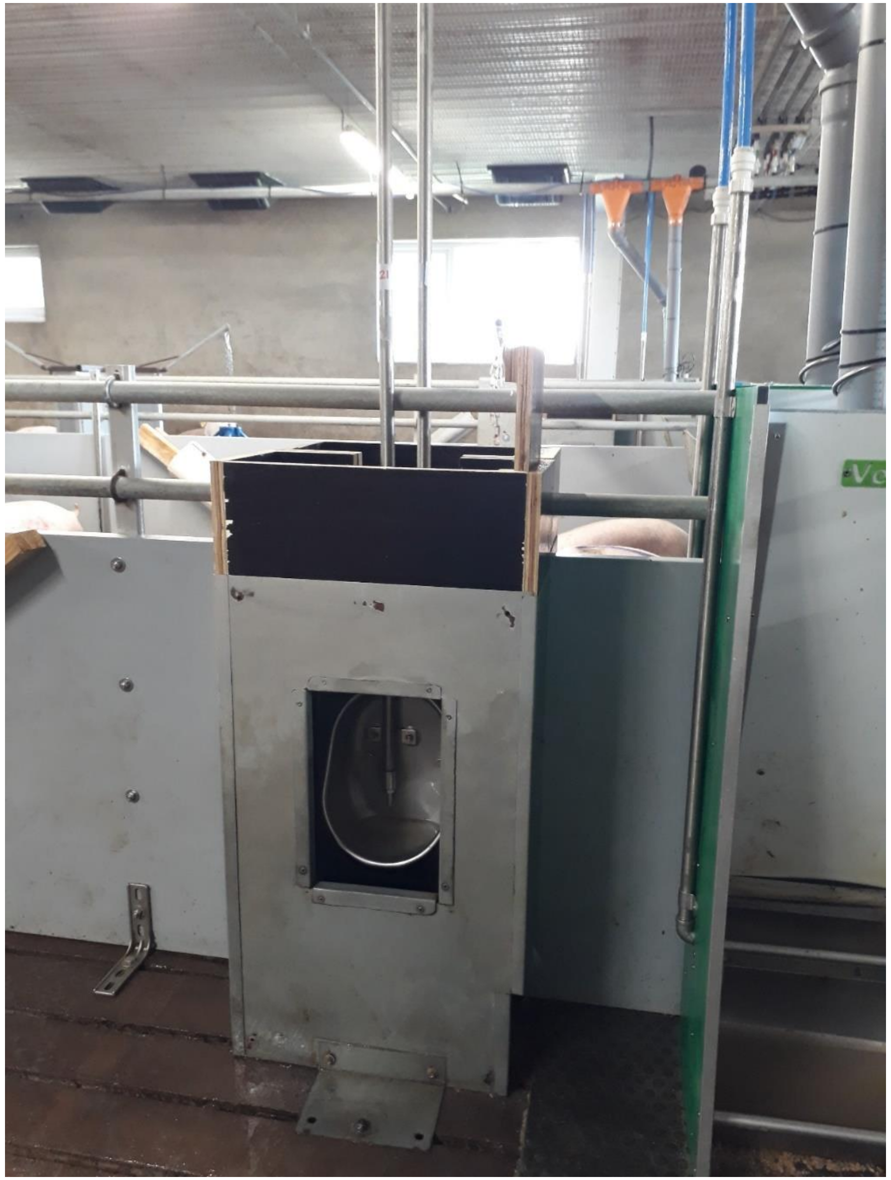

Supplementary figure S3: Wooden box around the drinker used to collect the wasted water
